# Systematic Transcriptome Analysis Reveals the Inhibitory Function of Cinnamaldehyde in Non-Small Cell Lung Cancer

**DOI:** 10.3389/fphar.2020.611060

**Published:** 2021-02-09

**Authors:** Ru Chen, Juan Wu, Chang Lu, Ting Yan, Yu Qian, Huiqing Shen, Yujing Zhao, Jianzhen Wang, Pengzhou Kong, Xinri Zhang

**Affiliations:** ^1^ Department of Respiratory and Critical Care Medicine, The First Hospital of Shanxi Medical University, Taiyuan, China; ^2^ College of Animal Science, Shanxi Agricultural University, Taigu, China; ^3^ Department of Pathology and Shanxi Key Laboratory of Carcinogenesis and Translational Research on Esophageal Cancer, Shanxi Medical University, Taiyuan, China

**Keywords:** cinnamaldehyde, long non-coding RNAs, micro RNAs, non-small cell lung cancer, messenger RNAs

## Abstract

Cinnamaldehyde (CA) is the main component extracted from the traditional Chinese medicine cinnamon. Recent studies revealed that CA has antiviral and anti-tumor effects. However, the effect and mechanism of CA on non-small cell lung cancer (NSCLC) through whole transcriptome sequencing integrated analysis have not been systematically investigated. In this study, whole transcriptome sequencing was used to identify differentially expressed messenger RNAs (mRNAs), micro RNAs (miRNAs), and long non-coding RNAs (lncRNAs) that were influenced by CA and screen regulatory pathways. The results showed that CA significantly inhibited proliferation, invasion, and migration, whereas it induced the apoptosis of NSCLC cells. CA inhibited tumor growth *in vivo*. Gene ontology and Kyoto Encyclopedia of Genes and Genomes analysis revealed that these differentially expressed mRNAs were potentially implicated in the CA-suppressing malignant phenotypes of NSCLC. According to the competing endogenous RNA (ceRNA) hypothesis, a ceRNA network was constructed, including 13 mRNAs, 6 miRNAs, and 11 lncRNAs. Kyoto Encyclopedia of Genes and Genomes analysis of the 13 mRNAs in the ceRNA network showed that suppressors of cytokine signaling 1 (SOCS1), BTG anti-proliferation factor 2 (BTG2), and Bruton tyrosine kinase (BTK) were significantly enriched in the JAK/STAT signaling pathway, RNA degradation, and nuclear factor-κB (NF-κB) signaling pathway related to cancer. These findings indicated that SOCS1, BTG2, and BTK play an essential role in CA against NSCLC. Meanwhile, based on the ceRNA network, three lncRNAs (long intergenic non-protein coding RNA 1504 [LINC01504], LINC01783, and THUMPD3 antisense RNA 1 [THUMPD3-AS1]) and three miRNAs (has-miR-155-5p, has-miR-7-5p, and has-miR-425-5p) associated with SOCS1, BTG2, and BTK may be important in CA against NSCLC. Taken together, the present study demonstrated the activity of CA against lung cancer and its potential use as a therapeutic agent.

## Introduction

Lung cancer is the most common type of cancer and the leading cause of cancer-related death. It is expected that its incidence rate will continue to increase (Chen et al., 2016). Approximately 85% of patients with lung cancer have non-small cell lung cancer (NSCLC). Lung adenocarcinoma and lung squamous cell carcinoma are the major types of NSCLC (Molina et al., 2008; Herbst et al., 2018). Although the development of drugs has greatly improved the therapy of patients with advanced NSCLC, the 5 years survival rates of these patients remain low (Minguet et al., 2016). Therefore, it is urgent to identify effective drugs for combating the malignant phenotype of lung cancer and elucidate the anti-tumor molecular mechanism.

Cinnamaldehyde (CA; C9H80, MW 132.16), the main component of the essential oil isolated from cinnamon, is a traditional Chinese medicine (Wu et al., 2017). Studies have demonstrated that CA can exert significant anti-cancer effects through multiple mechanisms. In human hepatocarcinoma cells, CA induces cell apoptosis by downregulating the expression of BCL-(XL) and upregulating that of CD95 and p53 (Ng and Wu 2011). Moreover, CA exerts an effective chemo-preventive effect by activating the JNK, ERK, and AKT signaling pathways, resulting in NRF2 nuclear translocation, eventually upregulating the expression of the phase II enzyme (Huang et al., 2011). CA could induce apoptosis and inhibit invasion and adhesion in colorectal cancer cells by antagonizing the activation of the PI3K/AKT signaling pathway (Li et al., 2016). In NSCLC, CA could induce apoptosis and reverse epithelial-mesenchymal transition through inhibition of the Wnt/β-catenin signaling pathway (Wu et al., 2017). In addition, CA could induce cell apoptosis by a novel circular RNA hsa_circ_0043256 (Tian et al., 2017).

Noncoding RNAs (ncRNAs) comprise rRNAs and others that can be further classified into short ncRNAs (micro RNAs [miRNAs], small interfering RNAs, small nucleolar RNAs, transfer RNAs, and piwi-interacting RNAs) and long ncRNAs (lncRNAs) (Chan and Tay. 2018). miRNAs are an abundant class of small ncRNAs with 20–24 nucleotides (Wang et al., 2020), which negatively regulate the gene expression of messenger RNAs (mRNAs) and translation inhibition involved in cell death and cell proliferation (Ambros 2004). lncRNAs are longer than 200 nucleotides, and play essential roles in the proliferation, metastasis, drug sensitivity, and progression of tumors (Xie et al., 2019). In 2011, Salmena et al. proposed a regulatory mechanism between ncRNAs and mRNA, namely the competing endogenous RNA (ceRNA) hypothesis (Salmena et al., 2011). According to this hypothesis, miRNAs could regulate the expression of target mRNAs and ncRNAs by binding to the miRNA response elements. It has been reported that ncRNAs serve as miRNA sponges to decrease miRNA abundance, thus relieving the inhibitory effect of miRNA on downstream target mRNAs (Guttman et al., 2009; Prensner and Chinnaiyan. 2011; Salmena et al., 2011). An increasing body of evidence has demonstrated that the ncRNAs play an important role in multiple cancers, such as breast cancer (Fan et al., 2018), liver cancer (Yan et al., 2018), and lung cancer (Sui et al., 2016).

The aim of this study was to unveil the regulatory effect of CA in NSCLC and investigate its regulatory mechanism, as well as identify key mediator molecules for the effect of CA on NSCLC.

## Materials and Methods

### Cell Culture

A549 and NCI-H1650 cell lines (lung adenocarcinoma), and SK-MES-1 and NCI-H226 cell lines (lung squamous cell carcinoma) were purchased from the Type Culture Collection of the Chinese Academy of Sciences (Shanghai, China). Cancer cells were cultured in RPMI1640 supplemented with 10% fetal bovine serum (FBS) and 1% penicillin (100 U/ml)/streptomycin (100 μg/ml) at 37°C with 5% CO_2_.

### Drugs and Reagents

The CA (purity 99.41% as measured by high-performance liquid chromatography) was purchased from the Shanghai BS Bio-Tech Co., Ltd (Shanghai, China) and dissolved in dimethyl sulfoxide. A total of 7.6 µL of CA was dissolved in 92.4 µL of dimethyl sulfoxide, and the final concentration of CA was 80 mg/ml. The 3-(4,5-Dimethylthiazol-2-thiazolyl)-2,5-diphenyltetrazolium bromide (MTT) was obtained from the Sigma Chemical Corporation (St. Louis, MO, United States). The annexin V/propidium iodide apoptosis detection kit was obtained from BD Biosciences (Franklin Lake, NJ, United States). The Matrigel was purchased from Corning (Wujiang, China). The TRIzol reagent was purchased from Invitrogen (Carlsbad, CA, United States). The RT reagent Kit and SYBR Green polymerase chain reaction (PCR) Master Mix were purchased from Promega (Madison, WI, United States). The primary antibodies against phospho-NF-κB (p-NF-κB) p65, *p*-JAK, phospho-signal transducer and activator of transcription 3 (p-STAT3), peroxisome proliferator-activated receptor gamma (PPARγ), and β-actin (1:1,000) were purchased from the Cell Signaling Technology Co., Ltd (Danvers, MA, United States).

### Cell Proliferation Assay

Cell viability was assessed using the MTT assay (Hao et al., 2020). Briefly, A549 (3,000 cells/well), NCI-H1650 (8,000 cells/well), SK-MES-1 (5,000 cells/well), and NCI-H226 (5,000 cells/well) cells were seeded in 96-well plates in the presence of various concentrations of CA. After treatment with CA for 24, 48, and 72 h, the cells were incubated with MTT (0.25 mg/ml) for 4 h at 37°C. Subsequently, the supernatant was discarded, and the formazan crystals were dissolved by adding dimethyl sulfoxide. The plates were analyzed at 490 nm to determine cell viability.

### Cell Apoptosis Assay

Cells were resuspended in 500 μl of binding buffer after treatment with various concentrations of CA for 24 h. Subsequently, 5 μl of annexin V-fluorescein isothiocyanate (FITC) and propidium iodide (PI) were added, and the cells were incubated for 15 min in the dark. Flow cytometry was employed to analyze the apoptosis of lung cancer cells.

### Cell Migration and Invasion Assay

For the migration assay, NSCLC cells were resuspended in 200 µL of FBS-free RPMI1640 medium that contained various concentrations of CA and placed in the upper chambers. The lower chambers were filled with 600 µL of RPMI1640 medium containing 10% FBS. At 24 h following the treatment with CA, the cells were fixed using 4% paraformaldehyde and stained with 0.25% crystal violet. The stained cells were imaged and counted to detect cell migration.

For the invasion assay, the membrane was coated with Matrigel (1:10 dilution) to form a matrix barrier. NSCLC cells were resuspended in 200 µL of RPMI1640 medium containing 5% FBS and various concentrations of CA and placed in the upper chambers. The lower chambers were filled with 600 µL of RPMI1640 medium containing 20% FBS. At 48 h after treatment with CA, the cells were fixed using 4% paraformaldehyde and stained with 0.25% crystal violet. The stained cells were imaged and counted to detect cell migration.

### *In vivo* Tumor Xenograft Experiments

Five-week-old female BALB/c nude mice were purchased from the Beijing Charles River Laboratory Animal Technology Co., Ltd. (Beijing, China) and used for the xenograft model. A549 cells were dissociated using trypsin and washed with sterilized phosphate-buffered saline (PBS). Subsequently, 0.1 ml of PBS containing 5 × 10^6^ cells was subcutaneously injected into the left flank of all mice. Mice were randomly separated into the vehicle group (PBS; *n* = 5) and CA group (100 mg/kg; *n* = 5). Both PBS and CA were intraperitoneally delivered once daily, and the mean tumor volumes were calculated using the following formula: volume = (length × width^2^)/2. The weight of mice was monitored and the tumor volume was measured once every 3 days. The mice were sacrificed 24 h after the last dose, and tumors were excised for weight and volume computation. All experiments were performed in accordance with the Guide for the Care and Use of Laboratory Animals, with the approval of Shanxi Medical University (Taiyuan, China).

### Whole Transcriptome Sequencing

A549 cells (control and 80 μg/ml CA groups) and SK-MES-1 cells (control and 40 μg/ml CA groups) were sent to Beijing Novogene (Beijing, China) to extract RNA, establish a cDNA library, and perform whole transcriptome sequencing. A total amount of 3 µg of RNA per sample was used as the input material for the preparation of the RNA samples. Sequencing libraries were generated using the NEBNext® Ultra™ RNA Library Prep Kit for Illumina® (NEB, United States). Gene expression was quantified using fragments per kilobase of transcript per million reads mapped (FPKM).

### Identification of Differentially Expressed (DE) Genes

The volcano plot was visualized using the R ggplot2 packages between the control and 80 μg/ml CA groups of the A549 cell line (including all genes), and the control and 40 μg/ml CA groups of the SK-MES-1 cell line (including all genes). Next, genes with an FPKM of any group >1, |log_2_FoldChange| >1, and adjusted *p-*value < 0.05 were considered as DE genes. The DE-mRNAs, DE-miRNAs, and DE-lncRNAs with the same expression trend intersecting from the 80 μg/ml CA group of A549 cells and the 40 μg/ml CA group of SK-MES-1 cells were regarded as common DE-mRNAs (CDE-mRNAs), CDE-miRNAs, and CDE-lncRNAs compared with control. Venn diagrams were plotted by VENNY 2.1.0 (https://bioinfogp.cnb.csic.es/tools/venny/index.html). The pheatmap package in R was used to plot the heatmap of the CDE-mRNAs, CDE-miRNAs, and CDE-lncRNAs.

### Functional Enrichment Analysis

Gene ontology (GO) functional enrichment was conducted using Metascape (https://metascape.org/gp/index.html) and Kyoto Encyclopedia of Genes and Genomes (KEGG) pathway enrichment analysis was performed using KOBAS 3.0 (http://kobas.cbi.pku.edu.cn/kobas3/genelist/) to investigate the possible functions of the CDE-mRNAs. The top 20 enriched GO categories and KEGG pathways were considered statistically significant and shown. The pathway. plot package in R was used to draw the GO term and KEGG pathway. The protein-protein interaction (PPI) network of the CDE-mRNAs was constructed to evaluate the interactive relationships by employing the STRING online database (https://string-db.org). The PPI pairs with a combined confidence score ≥0.4 were visualized in the network using the Cytoscape software.

### Prediction of miRNA-targeted mRNA and miRNA-mRNA network construction, prediction of miRNA-targeted lncRNA and ceRNA network construction

The lncRNA-miRNA-mRNA ceRNA network was constructed according to the miRNAs that can negatively regulate the expression of mRNAs and lncRNAs. Firstly, target mRNAs of the CDE-miRNAs were screened through the online mirtarbase (http://mirtarbase.cuhk.edu.cn/php/index.php) and Targetscan (http://www.targetscan.org/vert_72/). Only the miRNA-mRNA relationship pairs found in both databases were selected as candidate genes to construct the ceRNA network. Secondly, target lncRNAs of the CDE-miRNAs were screened through the online LncBase (http://carolina.imis.athena-innovation.gr/). Thirdly, the intersection of target mRNAs of the CDE-miRNAs and CDE-mRNAs was selected for further analysis. The intersection of target lncRNAs of the CDE-miRNAs and CDE-lncRNAs was also selected for further analysis. Finally, the miRNAs that were negatively regulated by the lncRNAs and mRNAs were selected to construct the miRNA-mRNA network and ceRNA network. Cytoscape (version 3.5.1) was used to visualize the miRNA-mRNA and lncRNA-miRNA-mRNA ceRNA networks.

### Validation by Real-Time PCR

Total RNA was extracted with TRIzol and converted to cDNA according to the instructions provided by the manufacturer. The cDNA was subjected to quantitative real-time PCR to detect mRNA expression using the GoTaq one-step real-time PCR kit with SYBR green; glyceraldehyde-3-phosphate dehydrogenase was used as an internal control. The gene primer list is shown in Table 1.

**TABLE 1:** The list of gene primer.

gene	Forward primer	Reverse primer
CREBRF	GTC​TCC​GAC​AAC​TTG​GGT​GAA​CAG	GCC​GAA​TCC​TTC​ATC​ATG​GTC​CTC
BTK	CCC​TGA​GCT​CAT​TAA​CTA​CCA​T	CCC​ATA​CTT​CAC​TAC​CCC​AAA​T
MXD1	ACA​AGG​ACA​GAG​ATG​CCT​TAA​A	TAA​ACT​CAA​CGT​AGT​GTG​TCG​A
BTG2	CAC​TCA​CAG​AGC​ACT​ACA​AAC​A	CAT​CTT​GTG​GTT​GAT​GCG​AAT​G
SOCS1	ACA​CGC​ACT​TCC​GCA​CAT​T	TAG​AAT​CCG​CAG​GCG​TCC​A
LINC01504	GAG​AGC​GTG​GCT​TTA​ACG​TCT	TCC​CTG​GCC​CAA​GCT​ATC​TC
LINC01783	CCA​ACA​AGG​ACA​GCA​GGT​GG	GTG​CGC​AAG​TGC​TTG​GTA​GA
LINC01484	GCC​TTA​GTG​CTG​CCA​TGC​TGA​G	GTG​CCT​GAT​GAG​TCC​TGG​GAA​ATG
LUCAT1	CAC​CAC​ACC​CAG​GAA​TCC​AAC​TTG	GTA​CAG​GCA​CGC​TAA​GTC​TCA​TCC
THUMPD3-AS1	GAG​ACA​AGC​CCG​ACC​TGC​TA	CTC​TGT​GCT​TAC​GCA​ACG​GAT​A

### Western Blotting Analysis

Total protein from cells was extracted using cell lysis buffer supplemented with protease inhibitors and phosphatase inhibitors. Equal amounts of protein were separated on 10% sodium dodecyl sulfate-polyacrylamide gel electrophoresis gels and transferred to nitrocellulose filter membranes. The membranes were blocked with 5% skim milk to block antigens at room temperature for 1 h and probed overnight at 4°C with primary antibodies. This was followed by further incubation with fluorescent secondary antibody for 2 h at room temperature. After washing, proteins were visualized with Odyssey (Licor, United States). The quantitative analysis was performed using the ImageJ software (National Institute of Mental Health, Bethesda, MD, United States).

### Statistical Analysis

One-way ANOVA followed by Fisher’s least significant difference (LSD) or Dunnett’s T3 were used to evaluate the differences between the groups when more than two groups and the independent sample *t*-test was used to analysis the differences of two groups using the SPSS 26.0 software (SPSS Inc., Chicago, IL, United States). Bioinformatics analysis was conducted using the aforementioned bioinformatics tools.

## Results

### CA Suppresses NSCLC Cell Proliferation

**FIGURE 1:**
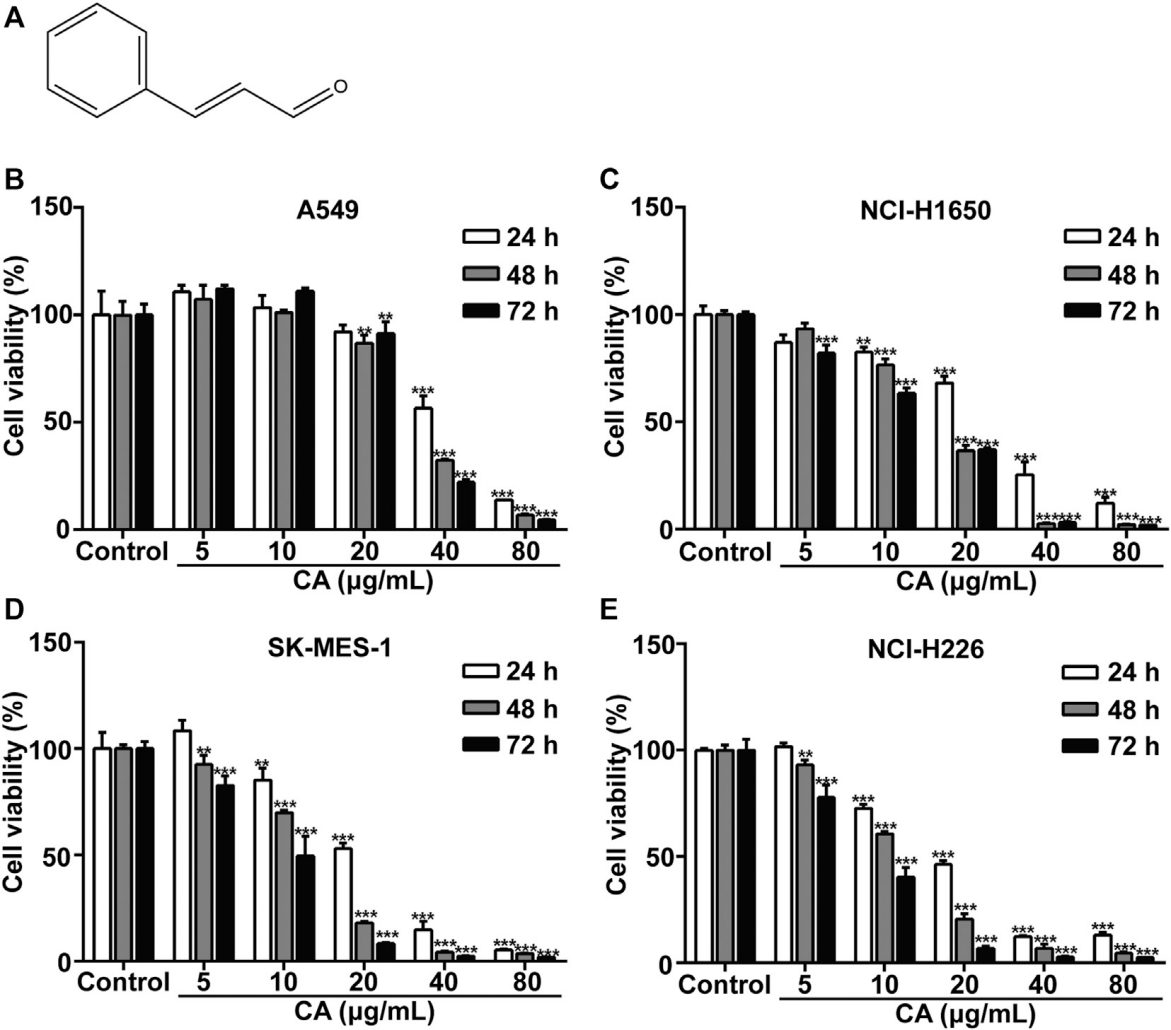
Cinnamaldehyde (CA) inhibits the proliferation of NSCLC cells. **(A)** Chemical structure of CA. **(B–E)** A549, NCI-H1650, SK-MES-1, and NCI-H226 cells were treated with various concentrations of CA for 24, 48, and 72 h, cell proliferation was measured using the MTT assay. Data are expressed as the mean ± SD of three independent experiments. **p < 0.01 and ***p < 0.001 vs. the control group. NSCLC, non-small cell lung cancer; SD, standard deviation.

The chemical structure of CA is shown in Figure 1A. A549, NCI-H1650, SK-MES-1, and NCI-H226 cell viability was significantly inhibited after treatment with CA for 24, 48, and 72 h. The observed inhibition rates were both dose- and time-dependent (Figure 1B–E). The IC_50_ values of the A549, NCI-H1650, SK-MES-1, and NCI-H226 cells were 47.44, 23.37, 27.63, and 28.13 μg/ml after treatment with CA for 24 h, respectively. According to the IC_50_ values, the 24 h treatment timepoint was selected for subsequent experiments.

### CA Induces Apoptosis and Inhibits Invasion and Migration in NSCLC Cells

**FIGURE 2:**
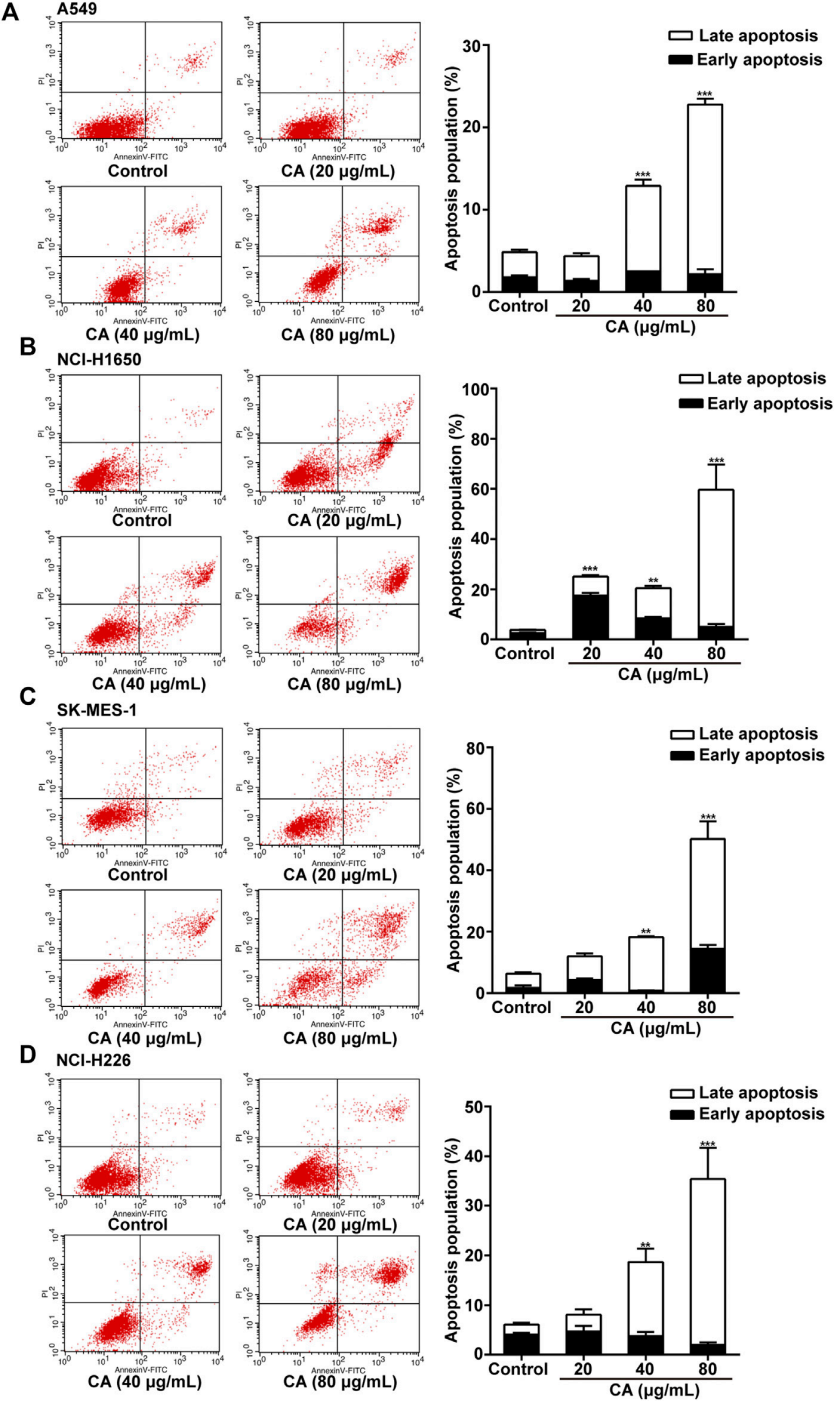
Cinnamaldehyde (CA) induces the apoptosis of NSCLC cells. **(A)** A549 cells were treated with 20, 40, and 80 μg/ml of CA for 24 h and stained with FITC-conjugated annexin V and PI to detect cell apoptosis through flow cytometry. **(B–D)** NCI-H1650, SK-MES-1, and NCI-H226 cells were treated with 10, 20, and 40 μg/ml of CA for 24 h and stained with FITC-conjugated annexin V and PI to detect cell apoptosis through flow cytometry. The quantitative results are shown in the right panel, and data are expressed as the mean ± SD of three independent experiments. **p < 0.01 and ***p < 0.001 vs. the control group. FITC, fluorescein isothiocyanate; NSCLC, non-small cell lung cancer; PI, propidium iodide; SD, standard deviation.

Annexin V-FITC analysis was performed to detect the apoptosis of cells after treatment with CA for 24 h. CA significantly induced apoptosis of A549, NCI-H1650, SK-MES-1, and NCI-H226 cells in a dose-dependent manner (Figure 2A–D). The results suggested that CA may suppress NSCLC cell proliferation via induction of apoptosis.

**FIGURE 3:**
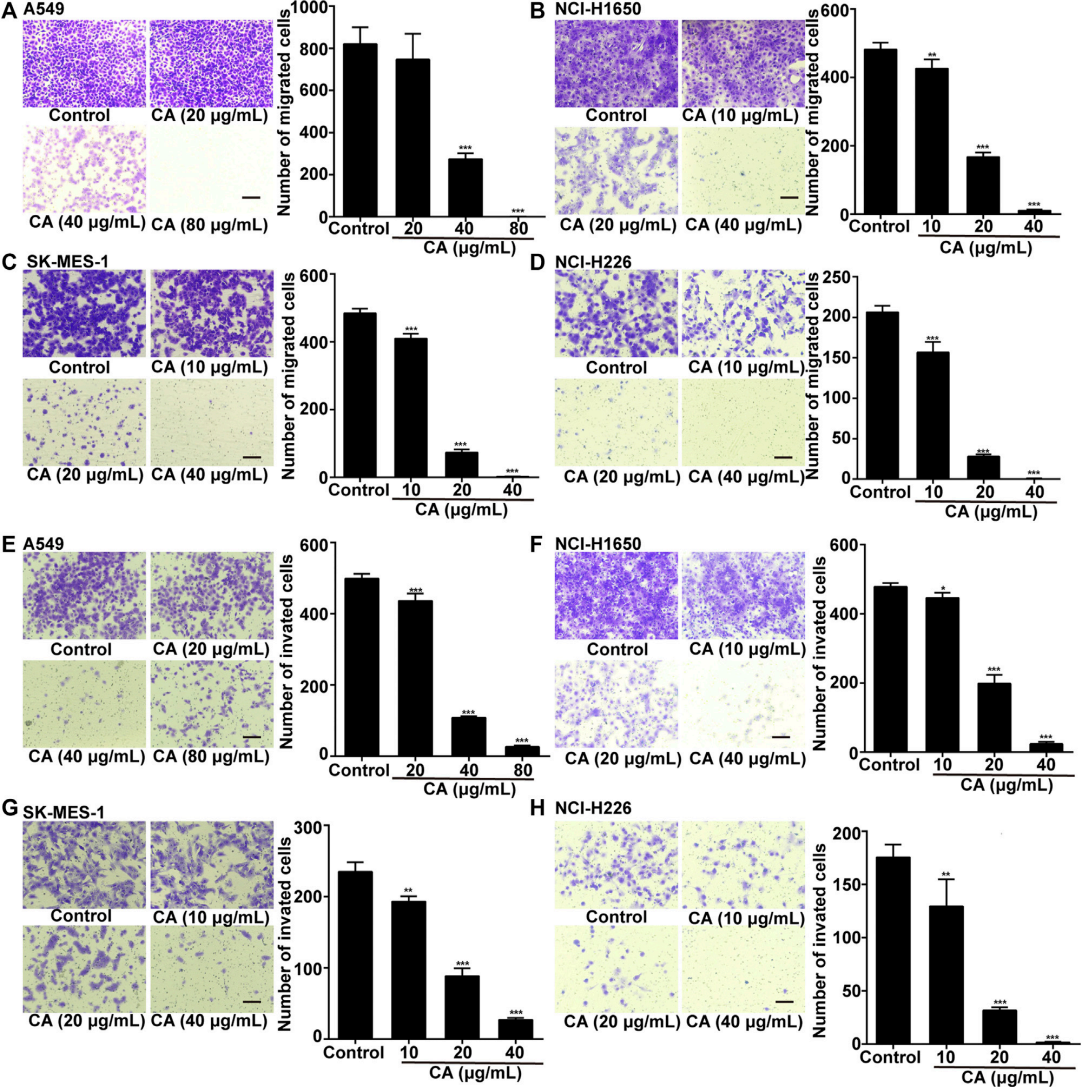
Cinnamaldehyde (CA) inhibits the invasion and migration of NSCLC cells. **(A)** A549 cells were treated with 20, 40, and 80 μg/ml of CA for 24 h to detect cell migration. **(B–D)** NCI-H1650, SK-MES-1, and NCI-H226 cells were treated with 10, 20, and 40 μg/ml of CA for 24 h to detect cell migration; **(E)** A549 cells were treated with 20, 40, and 80 μg/ml of CA for 48 h to detect cell invasion; **(F–H)** NCI-H1650, SK-MES-1, and NCI-H226 cells were treated with 10, 20, and 40 μg/ml of CA for 48 h to detect cell invasion. The quantitative results are shown in the right panel, and data are expressed as the mean ± SD of three independent experiments. *p < 0.05, **p < 0.01, and ***p < 0.001 vs. the control group. NSCLC, non-small cell lung cancer; SD, standard deviation.

To clarify whether CA could inhibit the invasion and migration of NSCLC cells, we conducted Transwell invasion and migration assays. The results showed that CA significantly inhibited cell invasion and migration in a dose-dependent manner (Figure 3A–H).

### CA Inhibits Tumor Growth in Mice

**FIGURE 4:**
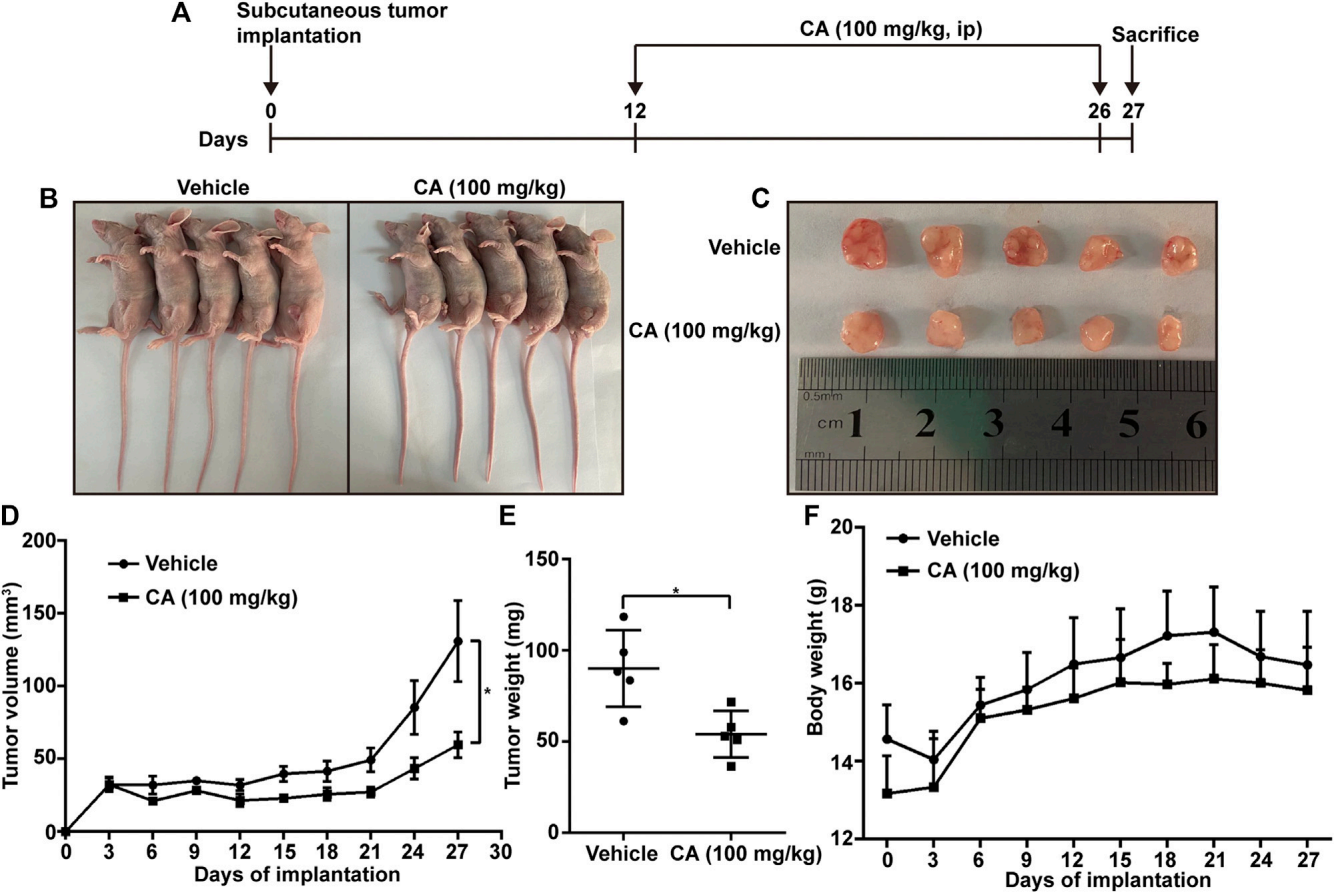
Cinnamaldehyde (CA) inhibits tumor growth in mice. **(A)** Diagram of the animal study protocol. **(B)** A549 cells (5 × 10^6^) were implanted into nude mice (*n* = 5). Representative images show the tumor xenografts. **(C,D)** Tumor volumes and weight were measured, and the volume of the tumors was calculated ((length × width^2^)/2). **(E)** Body weight was calculated every 3 days after implantation. Data are expressed as the mean ± SD of five independent experiments. *p < 0.05 vs. the vehicle group. ip, intraperitoneal injection; SD, standard deviation.

To study the effect of CA *in vivo*, a model of subcutaneous tumor implantation was established. The detailed protocol is shown in Figure 4A. The volume and weight of the tumors of mice treated with CA were smaller than those observed in the vehicle group (Figures 4B–E). In addition, there were no significant differences in body weight between CA-treated mice and vehicle mice (Figure 4F), indicating that CA may not induce physiological toxicity at the tested dose. The results of this experiment suggesting that CA could inhibit tumor growth.

### Identification of CDE-mRNAs, CDE-miRNAs, and CDE-lncRNAs

To elucidate the anti-cancer mechanism of CA, we used doses of 80 μg/ml and 40 μg/ml to treat A549 and SK-MES-1 cells, respectively. Subsequently, we carried out whole transcriptome sequencing to analyze the gene expression patterns in the CA-treated and -untreated cells.

**FIGURE 5:**
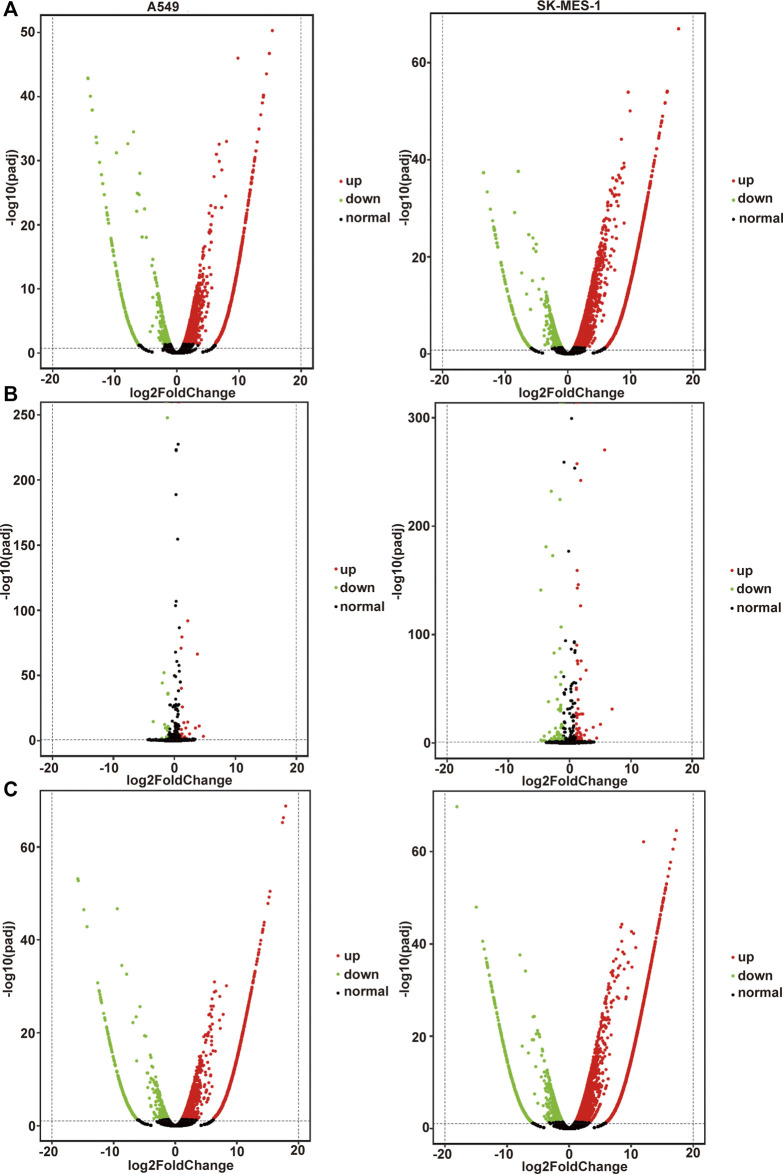
All genes affected by cinnamaldehyde (CA). **(A–C)** Volcano plot showing all altered mRNAs, miRNAs, and lncRNAs in A549 cells and SK-MES-1 cells. A549 cells are shown on the left, SK-MES-1 cells are shown on the right. mRNAs, messenger RNAs; miRNAs, micro RNAs; lncRNAs, long noncoding RNAs.

**FIGURE 6:**
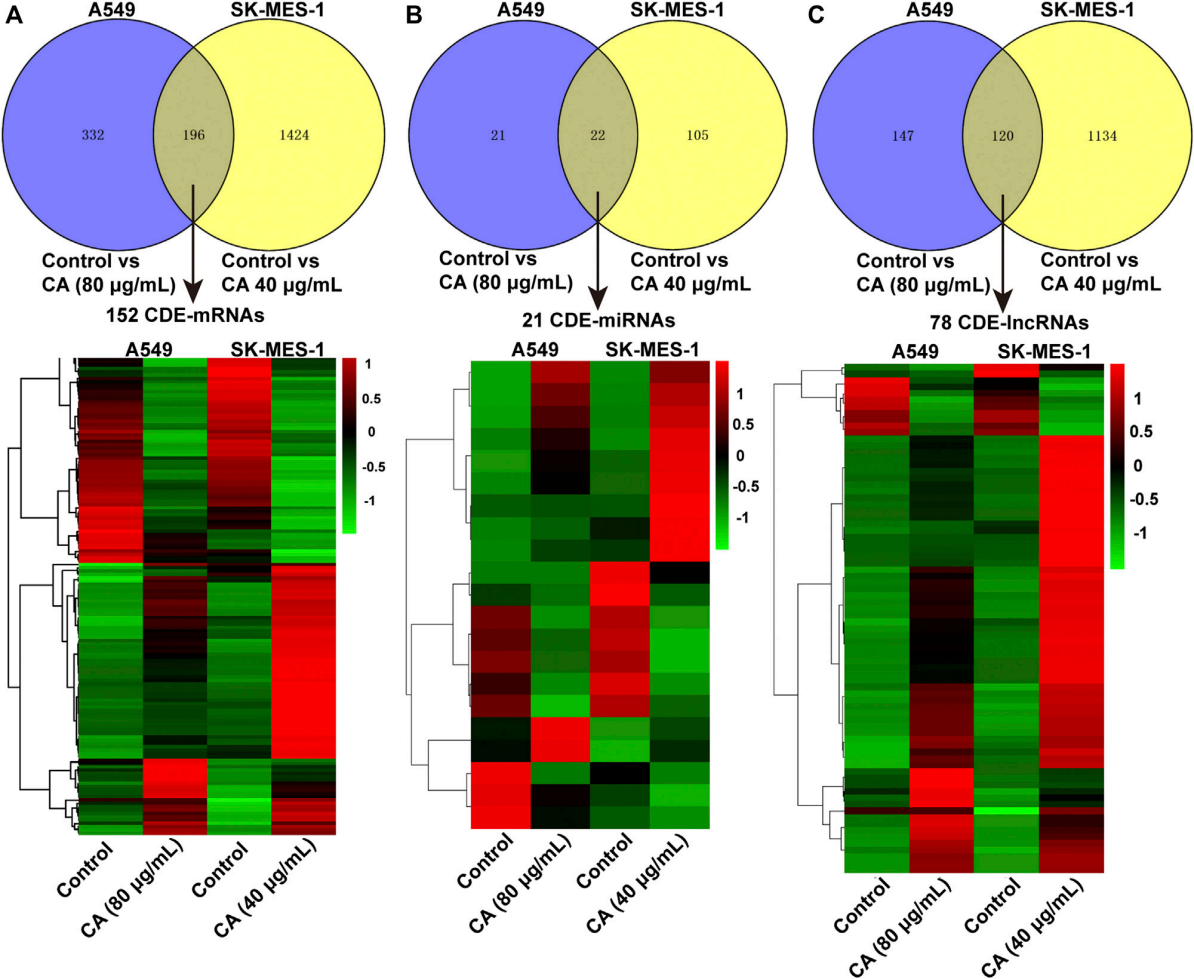
The expression profiles of DE-mRNAs were influenced by cinnamaldehyde (CA). **(A–C)** Venn diagram showing CDE-mRNAs, CDE-miRNAs, and CDE-lncRNAs in A549 cells and SK-MES-1 cells. Heatmap of CDE-mRNAs, CDE-miRNAs, and CDE-lncRNAs in A549 cells and SK-MES-1 cells. DE, differentially expressed; CDE, common differentially expressed; lncRNAs, long noncoding RNAs; mRNAs, messenger RNAs; miRNAs, micro RNAs.

The volcano plot displayed all altered mRNAs, miRNAs, and lncRNAs in A549 and SK-MES-1 cells (Figures 5A–C). As shown in the Venn diagram, 528 and 1,620 DE-mRNAs (Figure 6A), 43 and 127 DE-miRNAs (Figure 6B), and 267 and 1,254 DE-lncRNAs (Figure 6C) were identified in A549 and SK-MES-1 cells, respectively. Furthermore, 152 CDE-mRNAs (82 upregulated and 70 downregulated), 21 CDE-miRNAs (11 upregulated and 10 downregulated), and 78 CDE-lncRNAs (67 upregulated and 11 downregulated) were detected in two cell line (Figures 6A–C and Supplementary Table S1). The heatmap of CDE-mRNAs, CDE-miRNAs, and CDE-lncRNAs showed that after treatment with CA, gene expression was significantly changed compared with the control group (Figures 6A–C).

### Functional Analysis of the CDE-mRNAs

**FIGURE 7:**
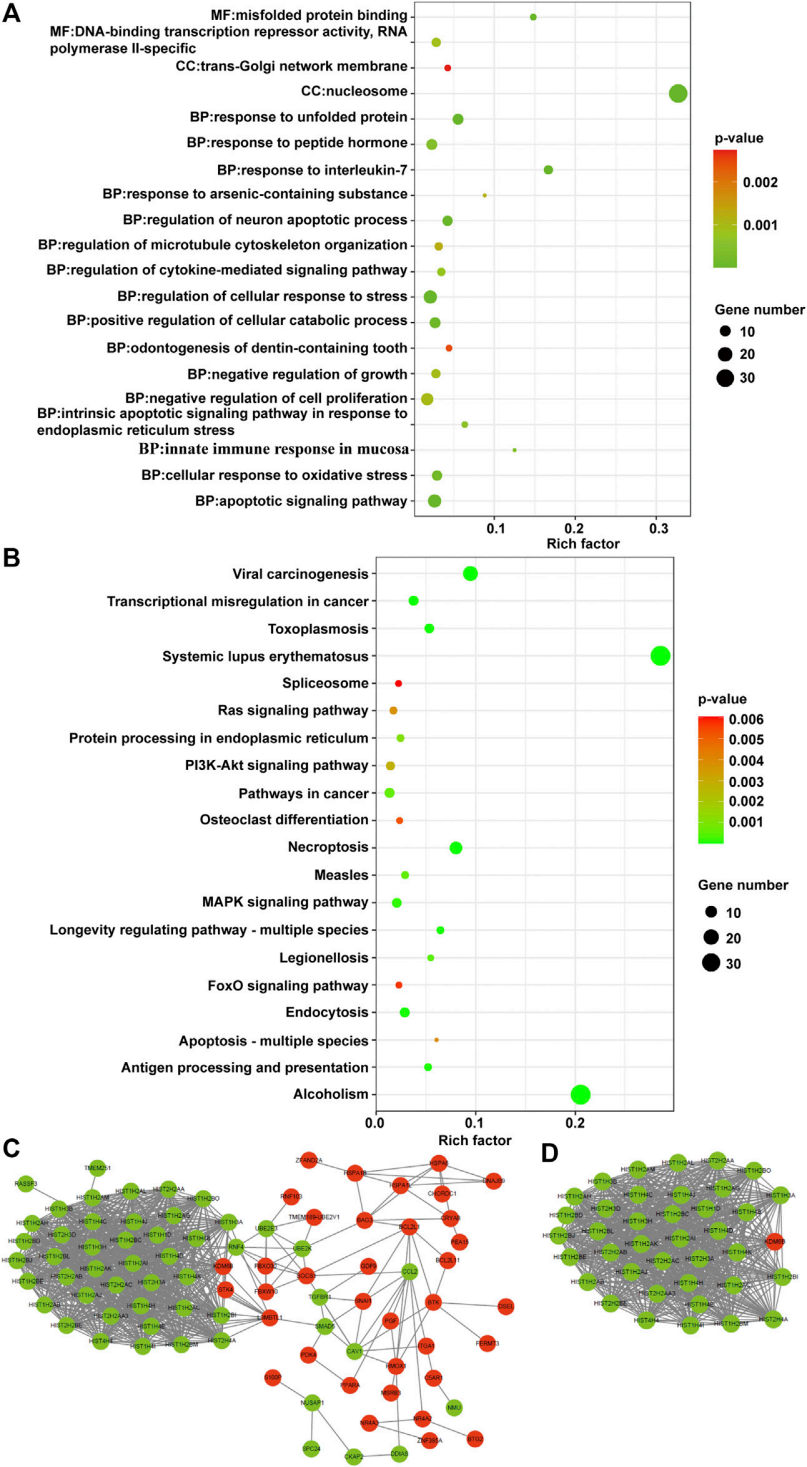
Functional analysis of the CDE-mRNAs. **(A)** The top 20 GO enrichment terms. **(B)** The top 20 KEGG enrichment terms. **(C)** The PPI network contains 88 nodes and 830 edges. **(D)** The subnetwork from the PPI network contains 39 nodes and 732 edges. The genes in red and green represent upregulation and downregulation, respectively. CDE-mRNAs, common differentially expressed-messenger RNAs; GO, Gene ontology; KEGG, Kyoto Encyclopedia of Genes and Genomes; PPI, protein-protein interaction.

To investigate the biological functions of the identified CDE-mRNAs, we performed GO term enrichment analysis and KEGG pathway analysis. The result of GO annotation indicated that CDE-mRNAs were significantly enriched in terms associated with cell apoptosis and proliferation, such as the apoptotic signaling pathway, the regulation of the neuron apoptotic process, the intrinsic apoptotic signaling pathway in response to endoplasmic reticulum stress, the negative regulation of growth, and the negative regulation of cell proliferation (Figure 7A). KEGG pathway analysis showed that CDE-mRNAs were significantly enriched in some cancer-associated pathways, including transcriptional misregulation in cancer, the MAPK signaling pathway, pathways in cancer, the PI3K/AKT signaling pathway, the Ras signaling pathway, apoptosis - multiple species, and the FOXO signaling pathway (Figure 7B). Taken together, these results indicated that CDE-mRNAs were closely related to apoptosis and cancer. To explore the relationship between these CDE-mRNAs, the PPI network was constructed using the STRING online database and visualized using Cytoscape (Figure 7C). The subnetwork (highly correlated module) was extracted from the whole PPI network using the Molecular COmplex DEtection (MCODE) algorithm. Highly correlated module analysis showed that CA affected histone genes, indicating that CA may play a central role in the regulation of transcription, DNA repair, DNA replication, and chromosomal stability (Figure 7D).

### Target mRNAs of CDE-miRNAs and Construction of the miRNA-mRNA Network

**FIGURE 8:**
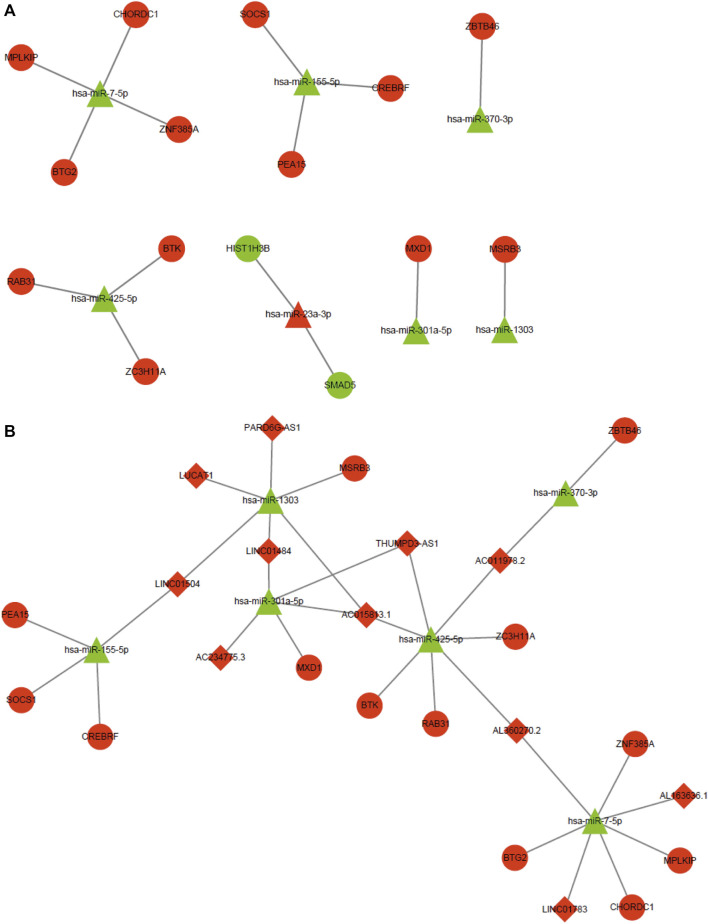
MiRNA-mRNA regulatory network and ceRNA network. **(A)** miRNA-mRNA regulatory network. Red and green represent upregulation and downregulation, respectively. Triangles and circles represent CDE-miRNAs and CDE-mRNAs, respectively. **(B)** ceRNA network. Red and green represent upregulation and downregulation, respectively. Triangles, circles, and diamonds represent CDE-miRNAs, CDE-mRNAs, and CDE-lncRNAs, respectively. CDE, common differentially expressed; ceRNA, competing endogenous RNA; lncRNAs, long noncoding RNAs; mRNAs, messenger RNAs; miRNAs, micro RNAs.

To further investigate the anti-cancer molecular mechanism involved in the effect of CA, target genes of CDE-miRNAs were predicted using the miRTarBase and Targetscan databases. A total of 24 mRNAs, such as a suppressor of cytokine signaling 1 (SOCS1), CREB3 regulatory factor (CREBRF), Bruton tyrosine kinase (BTK), BTG anti-proliferation factor 2 (BTG2), zinc finger and BTB domain containing 46 (ZBTB46), and M-phase specific PLK1 interacting protein (MPLKIP) overlapped with the CDE-mRNAs (Supplementary Table S2). According to the inverse regulatory relationship between miRNA and its target gene, a regulated miRNA-mRNA network was constructed using Cytoscape, which included 7 CDE-miRNAs and 15 target CDE-mRNAs (Figure 8A). Those CDE-miRNAs were selected for further analysis.

### Target lncRNAs of CDE-miRNAs and Construction of the ceRNA Regulatory Network

It is widely acknowledged that lncRNA can function as a sponge to competitively bind to miRNA. Hence, we predicted upstream lncRNAs that could potentially bind to those seven key CDE-miRNAs using an online LncBase database. A total of 14 lncRNAs, such as PARD6G antisense RNA 1 (PARD6G-AS1), BNC2-AS1, THUMPD3-AS1, lung cancer associated transcript 1 (LUCAT1), AC015813.1, long intergenic non-protein coding RNA 1504 (LINC01504), and LINC01484 overlapped with the CDE-lncRNAs (Supplementary Table S3). Based on the ceRNA hypothesis, the miRNAs were negatively regulated by the lncRNAs and mRNAs. The ceRNA network was constructed, including 6 CDE-miRNAs, 13 target CDE-mRNAs, and 11 target CDE-lncRNAs (Figure 8B).

### KEGG Analysis of the mRNAs in the ceRNA Network

**FIGURE 9:**
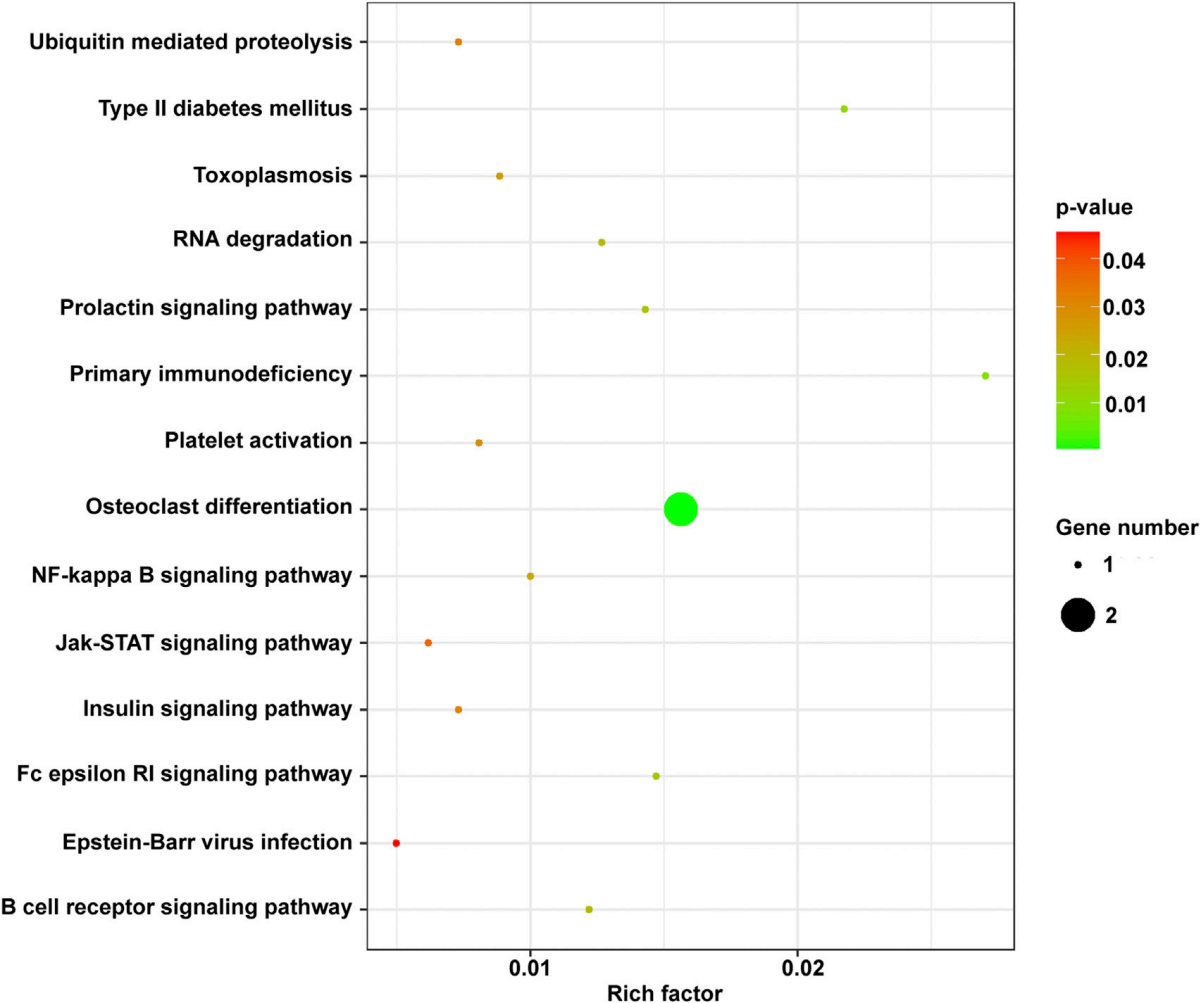
The KEGG enrichment terms of mRNAs in the ceRNA network. ceRNA, competing endogenous RNA; KEGG, Kyoto Encyclopedia of Genes and Genomes; mRNA, messenger RNA.

The KEGG pathway analysis showed that mRNAs in the ceRNA network were significantly enriched in the JAK/STAT signaling pathway (Owen et al., 2019), RNA degradation (Chang and Huang 2019), and NF-κB signaling pathway (Khan et al., 2020) related to cancer. SOCS1, BTK, and BTG2 were significantly enriched in these pathways (Figure 9).

Among lncRNAs in the ceRNA, LINC01504, LINC01783, and THUMPD3-AS1 were related to prognosis, indicating their important role in the development of cancer. Thus, based on the ceRNA network, three key lncRNAs (LINC01504, LINC01783, and THUMPD3-AS1) and miRNAs (has-miR-155–5p, has-miR-7-5p, and has-425–5p) that regulate SOCS1, BTG2, and BTK were identified.

### Gene Expression Verification via Quantitative PCR (qPCR)

**FIGURE 10:**
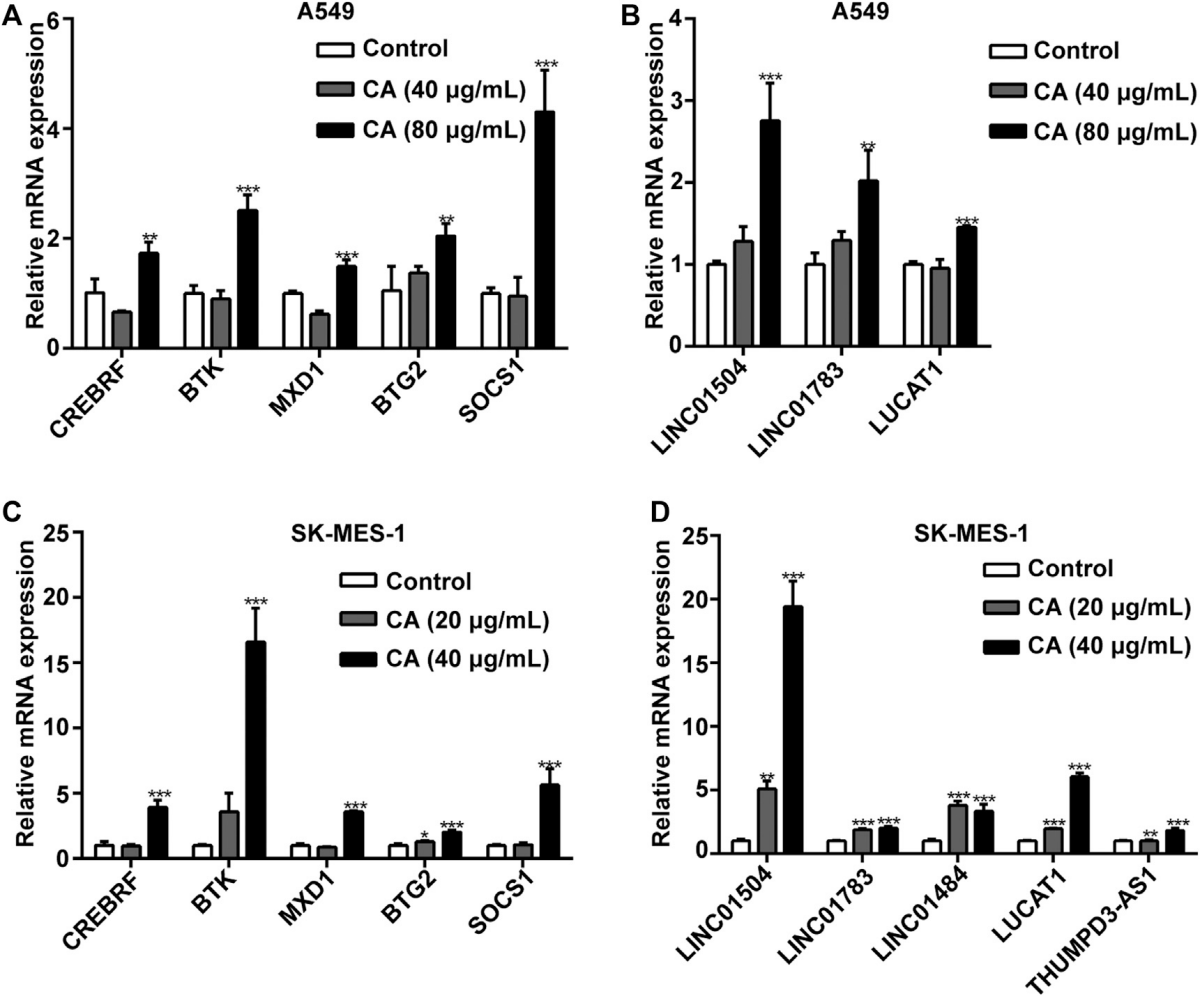
The effects of cinnamaldehyde (CA) on the expression of CDE-mRNAs and CDE-lncRNAs in the ceRNA network. **(A, B)** After treatment with CA for 24 h, the expression of CDE-mRNAs and CDE-lncRNAs in A549 cells was determined by RT-qPCR. **(C, D)** After treatment with CA for 24 h, the expression of CDE-mRNAs and CDE-lncRNAs in SK-MES-1 cells was determined by RT-qPCR. Data are expressed as the mean ± SD of three independent experiments. **p < 0.01 and ***p < 0.001 vs. the control group. CDE, common differentially expressed; ceRNA, competing endogenous RNA; lncRNAs, long noncoding RNAs; mRNAs, messenger RNAs; RT-qPCR, reverse transcription-quantitative polymerase chain reaction; SD, standard deviation.

We also determined the accuracy and reliability of the present bioinformatics analysis. For this purpose, qPCR was used to evaluate gene expression in the A549 and SK-MES-1 cells. Genes in the ceRNA network were selected to verify the reliability of the sequencing results. Consistent with the sequencing results, the expression levels of five mRNAs (i.e., SOCS1, CREBRF, MAX dimerization protein 1 [MXD1], BTK, and BTG2), and three lncRNAs (i.e., LINC01504, LINC01783, and LUCAT1) were significantly elevated after the A549 cells were treated with CA (Figures 10A). The expression levels of five mRNAs (i.e., SOCS1, CREBRF, MXD1, BTK, and BTG2) and five lncRNAs (i.e., LINC01504, LUCAT1, LINC01484, THUMPD3-AS1, and LINC01783) were significantly elevated after the SK-MES-1 cells were treated with CA (Figures 10C).

### CA inhibits the JAK/STAT3 signaling pathway, NF-κB signaling pathway, and RNA degradation signaling pathway

**FIGURE 11:**
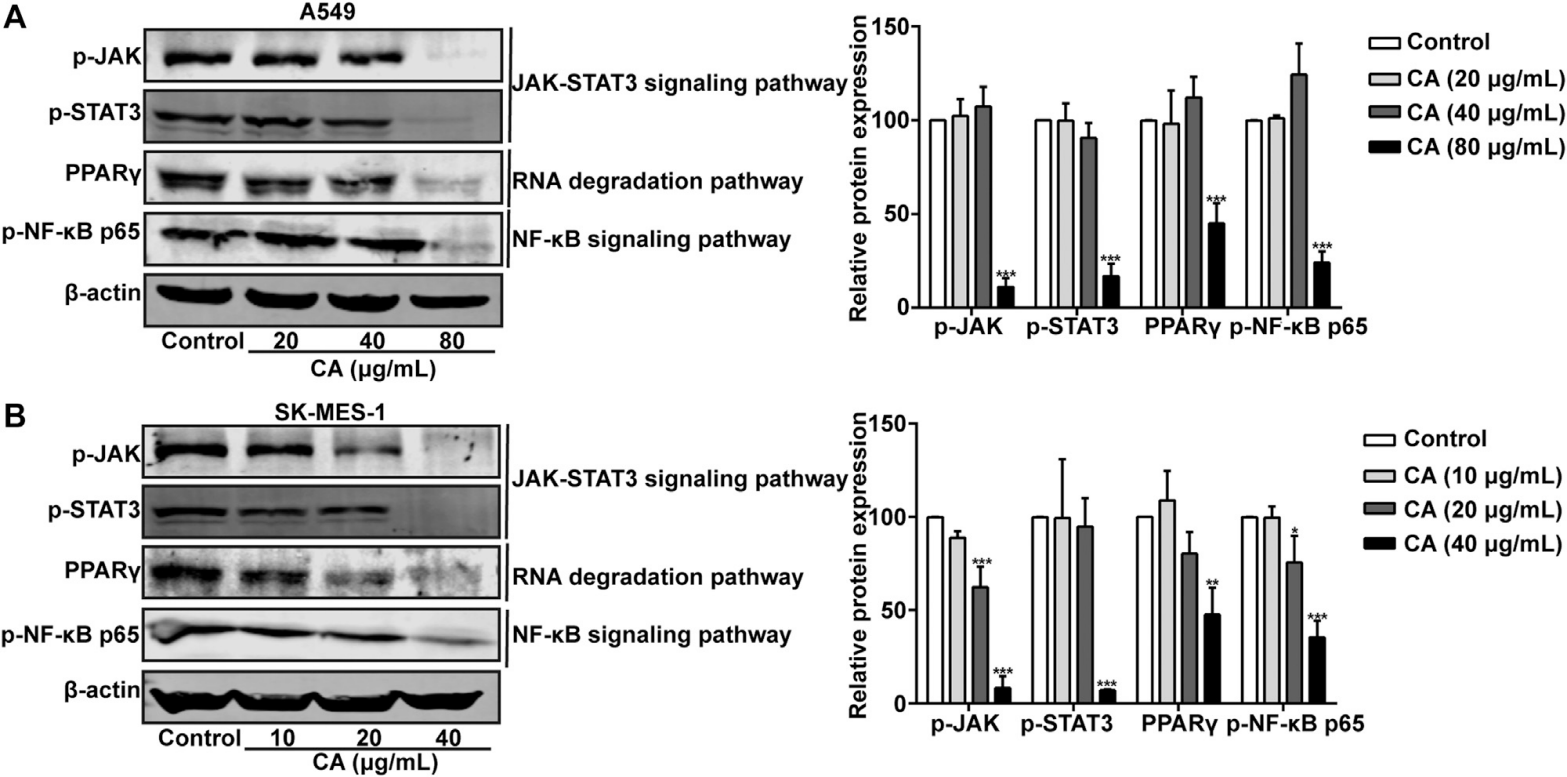
Cinnamaldehyde (CA) inhibits the activation of the JAK/STAT3 signaling pathway, RNA degradation, and NF-κB signaling pathway. **(A)** A549 cells were treated with 20, 40, and 80 μg/ml of CA for 24 h. The protein expression of p-JAK, p-STAT3, PPARγ, and p-NF-κB p65 was determined using western blotting and the quantitative results are shown in the right panel. **(B)** SK-MES-1 cells were treated with 10, 20, and 40 μg/ml of CA for 24 h. The protein expression of p-JAK, p-STAT3, PPARγ, and p-NF-κB p65 was determined using western blotting and the quantitative results are shown in the right panel. Data are expressed as the mean ± SD of three independent experiments. *p < 0.05, **p < 0.01, and ***p < 0.001 vs. the control group. NF-κB, nuclear factor-κB; p-JAK, phospho-JAK; p-STAT3, phospho-signal transducer and activator of transcription 3; PPARγ, peroxisome proliferator-activated receptor gamma; SD, standard deviation.

The involvement of key pathways, such as the JAK/STAT3 signaling pathway, NF-κB signaling pathway, and RNA degradation pathway, in the inhibitory effects of CA on NSCLC was analyzed. The phosphorylation levels of JAK, STAT3, and NF-κB p65 were significantly suppressed by CA in A549 and SK-MES-1 cells (Figures 11A,B). Moreover, the expression of PPARγ was also inhibited by CA (Figures 11A,B). These results suggested that CA could inhibit multiple pathways, including the JAK/STAT3, NF-κB, and RNA degradation signaling pathway.

## Discussion

Natural products against cancer have been considered in numerous studies. After experimental and clinical verification, agents such as vincristine, camptothecin, and paclitaxel have been approved for medical use and have become essential drugs for the treatment and prevention of tumors. Research has demonstrated that CA can exert an anti-tumor effect. In this study, it could inhibit proliferation, induce apoptosis, and inhibit the migration and invasion of NSCLC cells *in vitro*. Moreover, it could efficiently suppress NSCLC progression *in vivo*. In the present study, we used whole transcriptome sequencing to reveal the anti-tumor mechanism involved in the effects of CA and identify novel prognostic indicators of lung cancer.

In the present study, we successfully constructed an lncRNA-miRNA-mRNA regulatory network. Firstly, we screened 152 dysregulated CDE-mRNAs; GO analysis revealed that those CDE-mRNAs were significantly enriched in some cancer-related GO items, such as the regulation of the neuron apoptotic process (Hollville et al., 2019), the intrinsic apoptotic signaling pathway in response to endoplasmic reticulum stress (Ghobrial et al., 2005), the negative regulation of growth, and the negative regulation of cell proliferation (Giordano and Tommonaro 2019). Subsequent KEGG pathway enrichment analysis also highlighted various pathways involved in cancer progression, including transcriptional misregulation in cancer, the MAPK signaling pathway (Davis 2000), pathways in cancer, the PI3K/AKT signaling pathway (Martini et al., 2014), the Ras signaling pathway (Fang 2016), apoptosis across multiple species, and the FOXO signaling pathway (Farhan et al., 2017). The PPI network was constructed to exhibit complicated associations among these CDE-mRNAs. Therefore, these CDE-mRNAs interact with each other and may play important roles in the effect of CA on cancer.

The ceRNA network was constructed according to the expression analysis of sequencing data and the ceRNA hypothesis. Among the identified three-lncRNA signature, LINC01504 is involved in nontranslocation-related sarcomas (Delespaul et al., 2017). The specific anti-cancer mechanism of LINC01504 is unclear; however, it is lowly expressed in NSCLC and associated with the prognosis of NSCLC. A recent study reported that LINC01783 was associated with the proliferation, migration, and invasion of cervical cancer cells, indicating its important role in cancer (Chen et al., 2020). It was also discovered that THUMPD3-AS1 is involved in many tumors, and affects the proliferation and self-renewal of NSCLC cells (Hu et al., 2019). In our study, LINC01504 and SOCS1 in the ceRNA network were upregulated after treatment with CA, whereas has-miR-155–5p was downregulated. Moreover, CA could inhibit the activation of the JAK/STAT3 signaling pathway. Previous studies have shown that hsa-miR-155–5p functions as an oncogene to promote the progression of hepatocellular carcinoma (Fu et al., 2017) and affect cell proliferation and apoptosis (Zhao et al., 2019). SOCS1 could reduce inflammation within the tumor microenvironment to contribute to tumor suppression in a cancer cell-intrinsic manner (Villalobos-Hernandez et al., 2017). In addition, SOCS1 could inhibit cell growth and attenuate MET signaling, thereby inhibiting hepatocyte growth factor-induced migration and invasion (Flowers et al., 2005). After treatment with CA, LINC01783 and BTG2 in the ceRNA network were upregulated, whereas hsa-miR-7-5p was downregulated. Also, CA could modulate PPARγ expression in the RNA degradation pathway. In colorectal cancer, has-miR-7 is significantly upregulated and correlates with venous invasion, tumor depth, lymph node metastasis, lymphatic invasion, liver metastasis, and poor overall survival (Nagano et al., 2016). miR-7-5p was significantly upregulated in neuroendocrine neoplasms (Heverhagen et al., 2018). Inhibition of miR-7 expression could inhibit migration and proliferation, as well as induce apoptosis in renal cell carcinoma (Yu et al., 2013). BTG2 is a member of the BTG/TOB family, and its encoded proteins have antiproliferative properties. BTG2-encoded protein products have been associated with transcriptional regulation, DNA repair, cell division, and mRNA stability (Yuniati et al., 2019). After treatment with CA, THUMPD3-AS1 and BTK in the ceRNA network were upregulated, whereas hsa-miR-425–5p was downregulated. In addition, CA could inhibit the activation of the NF-κB signaling pathway. Downregulation of miR-425–5p expression inhibited the proliferation, invasion, and migration of gastric cancer cells (Yan et al., 2017). BTK is a non-receptor kinase that plays an essential role in the proliferation of numerous B cell malignancies and is also an important component of the tumor microenvironment (Pal Singh et al., 2018). Traditionally, BTK was considered oncogenic in B cell malignancies. However, recent data have shown that it can also act as a tumor suppressor in other types of cancer, as an essential member of the p53 and p73 damage response (Rada et al., 2018). The present findings indicate that LINC01504, LINC01783, and THUMPD3-AS1 may play roles as NSCLC suppressors.

The current study had some limitations. Firstly, we only focused on the negative regulation of miRNA-mRNA and miRNA-lncRNA of the ceRNA hypothesis and its prognostic value, which may exclude more complex regulatory mechanisms. Secondly, experiments should have been performed to verify the specific mechanism involved in the effects of CA on lung cancer, and experimental validation will be carried out in the future. However, this study aimed to build a regulatory ceRNA network and screen key lncRNAs that can provide a basis for further experimental and clinical studies.

## Conclusion

In summary, CA is effective against lung cancer. LINC01504, LINC01783, THUMPD3-AS1, has-miR-155–5p, has-miR-425–5p, and has-miR-7-5p may be key ncRNAs in the suppression of malignant phenotypes of NSCLC by CA. The JAK/STAT signaling pathway, RNA degradation, and NF-κB signaling pathway may be key regulatory pathways involved in the effect of CA against NSCLC. These data highlight CA as a potential therapeutic agent for the clinical treatment of lung cancer.

## Data Availability

The original contributions presented in the study are publicly available. This data can be found here: https://dataview.ncbi.nlm.nih.gov/object/PRJNA686481?reviewer=270idd6itbb0hvlr1siot1i4lh.
