# Supplementary material for: Systematic Transcriptome Analysis Reveals the Inhibitory Function of Cinnamaldehyde in Non-Small Cell Lung Cancer
Source: Front Pharmacol. 2021 Feb 9;11:611060. doi: 10.3389/fphar.2020.611060 (PMC7900626; doi:10.3389/fphar.2020.611060)
Supplement: Supplementary file 1 [file Table1.docx]

**Supplemental Table S1. The list of CDE-mRNA, CDE-miRNAs, CDE-lncRNAs**

| gene type | up regulation | down regulation |
| --- | --- | --- |
| CDE-mRNAs | PDK4  BTK  ZC3H11A  MXD1  CATSPERG  HMOX1  DHRS2  STK4  NECAB2  CRYM  CA2  RASD1  CRYAB  CHORDC1  RSRP1  FBXO30  NR4A3  PGF  TMEM189-UBE2V1  SNAI1  C1orf61  DNAJB9  CHAC1  ZBTB46  SESN2  GCH1  KDM6B  GIPC2  RPL3L  NFIC  RERE  PNLDC1  GCNA  FERMT3  BAG3  MR1  BCL2L11  NR4A2  DDAH1  WDR66  BTG2  ZNF385A  PEA15  SPDYA  S100P  GDF9  CREBRF  SYTL3  PPP1R36  SCG5  PIP5KL1  MPLKIP  RAB31  PHYHIP  DSEL  BCL2L1  FBXW10  RND1  HSPA6  MSRB3  SCUBE2  ZFAND2A  EFCAB13  R3HDM2  ZBTB20  DCAF4L1  TSPYL2  SOCS1  L3MBTL1  PPARA  ZKSCAN3  ZNF720  C5AR1  SYS1  HLA-DMA  NEU1  HSPA1B  HSPA1L  ITGA1  RNF103  CDRT1  AC004922.1 | EIF4B  RNF4  TRMT11  UBE2K  SEPHS1  PRPS2  CAV1  AP1S1  TGFBR1  CCL2  NMU  SMAD5  KIAA1191  HIST1H1D  HIST1H2BJ  MEA1  CKAP2  NUSAP1  ARHGAP29  RASSF3  TMEM251  HIST1H2BD  HIST1H4H  TOMM40L  SPC24  DDIAS  CASC4  UBE2E3  RAB43  HIST1H2AC  HIST1H2BC  HIST2H3D  ARSI  HIST2H2AC  HIST2H2AB  HIST2H2BE  HIST1H2BL  CCDC137  TCEA1  HIST1H2AI  HIST1H2AG  HIST1H4C  HIST1H4J  HIST4H4  HIST2H2AA3  HIST2H3A  TRAF3IP1  UBXN2B  CCNL2  ZBED6  HIST2H4A  HIST2H2AA4  HIST1H4K  HIST1H2BM  HIST1H3B  HIST1H2BE  HIST1H2BO  HIST1H2AH  HIST1H2AK  HIST1H3A  HIST1H4I  HIST1H2AJ  HIST1H2AL  HIST1H4E  HIST1H4D  HIST1H2AB  HIST1H2BI  HIST1H2AM  HIST1H4B  HIST1H3H |
| CDE-miRNA | hsa-miR-1246  hsa-miR-1307-5p  hsa-miR-147b-3p  hsa-miR-193a-3p  hsa-miR-23a-3p  hsa-miR-23b-3p  hsa-miR-320b  hsa-miR-320c  hsa-miR-320d  hsa-miR-330-5p  hsa-miR-503-5p | hsa-miR-1303  hsa-miR-155-5p  hsa-miR-16-2-3p  hsa-miR-27b-5p  hsa-miR-301a-5p  hsa-miR-370-3p  hsa-miR-425-5p  hsa-miR-7-5p  hsa-miR-7974  hsa-miR-9901  novel_765 |
| CDE-lncRNA | MIR31HG  LINC00324  LINC00862  THUMPD3-AS1  AL645941.1  LINC01504  AC074366.1  AL365436.2  IDI2-AS1  LINC01783  AC098617.1  LURAP1L-AS1  OLMALINC  AC099811.1  AC008443.2  LUCAT1  AC084082.1  AC090802.1  LINC01484  AP000442.1  AP000438.1  LBX2-AS1  AL163636.1  HIF1A-AS2  AC083843.3  AC051619.8  AC234775.3  AC012181.1  AC124068.2  AC011978.2  AC036108.3  AL360270.2  AC108134.2  AC015813.1  PARD6G-AS1  AP001160.3  AL136164.2  AC016831.5  AC092687.3  AL031775.1  AL135925.1  AC011912.1  AC004784.1  AC106858.1  AL592295.3  AL035461.3  XLOC_000579  XLOC_000635  XLOC_000686  XLOC_001014  XLOC_007156  XLOC_017884  XLOC_017888  XLOC_018192  XLOC_049060  XLOC_101073  XLOC_101269  XLOC_104796  XLOC_123388  XLOC_172219  XLOC_177890  XLOC_179807  XLOC_186728  XLOC_186747  XLOC_194139  XLOC_211338  XLOC_245788 | TGFB2-AS1  EZR-AS1  BNC2-AS1  PTPRJ-AS1  AP002360.1  AC034102.3  AL355075.4  AP003096.1  AC239868.2  Z93241.1  AL353759.1 |
