# Supplementary material for: Systematic Transcriptome Analysis Reveals the Inhibitory Function of Cinnamaldehyde in Non-Small Cell Lung Cancer
Source: Front Pharmacol. 2021 Feb 9;11:611060. doi: 10.3389/fphar.2020.611060 (PMC7900626; doi:10.3389/fphar.2020.611060)
Supplement: Supplementary file 2 [file Table2.docx]

**Supplemental Table S2 The list of miRNA-mRNA pair**

| miRNA | miRNA regulation | mRNA | mRNA regulation |
| --- | --- | --- | --- |
| hsa-miR-1246 | UP | CREBRF | UP |
| hsa-miR-1303 | DOWN | MSRB3 | UP |
| hsa-miR-155-5p | DOWN | SOCS1 | UP |
| hsa-miR-155-5p | DOWN | SMAD5 | DOWN |
| hsa-miR-155-5p | DOWN | CREBRF | UP |
| hsa-miR-155-5p | DOWN | PEA15 | UP |
| hsa-miR-16-2-3p | DOWN | UBE2E3 | DOWN |
| hsa-miR-193a-3p | UP | DNAJB9 | UP |
| hsa-miR-193a-3p | UP | DDAH1 | UP |
| hsa-miR-23a-3p | UP | HIST1H3B | DOWN |
| hsa-miR-23a-3p | UP | SESN2 | UP |
| hsa-miR-23a-3p | UP | SMAD5 | DOWN |
| hsa-miR-23b-3p | UP | SESN2 | UP |
| hsa-miR-23b-3p | UP | CA2 | UP |
| hsa-miR-27b-5p | DOWN | HIST1H2BD | DOWN |
| hsa-miR-301a-5p | DOWN | MXD1 | UP |
| hsa-miR-320b | UP | CREBRF | UP |
| hsa-miR-320c | UP | CREBRF | UP |
| hsa-miR-320d | UP | CREBRF | UP |
| hsa-miR-370-3p | DOWN | ZBTB46 | UP |
| hsa-miR-425-5p | DOWN | RAB31 | UP |
| hsa-miR-425-5p | DOWN | ZC3H11A | UP |
| hsa-miR-425-5p | DOWN | BTK | UP |
| hsa-miR-503-5p | UP | CREBRF | UP |
| hsa-miR-503-5p | UP | CHAC1 | UP |
| hsa-miR-7-5p | DOWN | BTG2 | UP |
| hsa-miR-7-5p | DOWN | CAV1 | DOWN |
| hsa-miR-7-5p | DOWN | UBXN2B | DOWN |
| hsa-miR-7-5p | DOWN | MPLKIP | UP |
| hsa-miR-7-5p | DOWN | CHORDC1 | UP |
| hsa-miR-7-5p | DOWN | ZNF385A | UP |
