# Supplementary material for: Systematic Transcriptome Analysis Reveals the Inhibitory Function of Cinnamaldehyde in Non-Small Cell Lung Cancer
Source: Front Pharmacol. 2021 Feb 9;11:611060. doi: 10.3389/fphar.2020.611060 (PMC7900626; doi:10.3389/fphar.2020.611060)
Supplement: Supplementary file 3 [file Table3.docx]

**Supplemental Table S3 The list of miRNA-lncRNA pair**

| miRNA | miRNA regulation | lncRNA | lncRNA regulation |
| --- | --- | --- | --- |
| hsa-miR-155-5p  hsa-miR-1303  hsa-miR-1303  hsa-miR-1303  hsa-miR-1303  hsa-miR-1303  hsa-miR-1303  hsa-miR-301a-5p  hsa-miR-301a-5p  hsa-miR-301a-5p  hsa-miR-301a-5p  hsa-miR-23a-3p  hsa-miR-23a-3p  hsa-miR-425-5p  hsa-miR-370-3p  hsa-miR-7-5p  hsa-miR-7-5p  hsa-miR-7-5p  hsa-miR-425-5p  hsa-miR-7-5p  hsa-miR-425-5p  hsa-miR-425-5p | DOWN  DOWN  DOWN  DOWN  DOWN  DOWN  DOWN  DOWN  DOWN  DOWN  DOWN  UP  UP  DOWN  DOWN  DOWN  DOWN  DOWN  DOWN  DOWN  DOWN  DOWN | LINC01504  AC239868.2  PARD6G-AS1  LUCAT1  LINC01504  LINC01484  AC015813.1  THUMPD3-AS1  AC234775.3  LINC01484  AC015813.1  THUMPD3-AS1  AC036108.3  AL360270.2  AC011978.2  BNC2-AS1  AL360270.2  LINC01783  AC011978.2  AL163636.1  AC015813.1  THUMPD3-AS1 | UP  DOWN  UP  UP  UP  UP  UP  UP  UP  UP  UP  UP  UP  UP  UP  DOWN  UP  UP  UP  UP  UP  UP |
